# A *Klebsiella pneumoniae* NDM-1+ bacteriophage: Adaptive polyvalence and disruption of heterogenous biofilms

**DOI:** 10.3389/fmicb.2023.1100607

**Published:** 2023-02-17

**Authors:** Eddie B. Gilcrease, Sherwood R. Casjens, Ananda Bhattacharjee, Ramesh Goel

**Affiliations:** ^1^Department of Civil and Environmental Engineering, University of Utah, Salt Lake City, UT, United States; ^2^School of Biological Sciences, University of Utah, Salt Lake City, UT, United States; ^3^Division of Microbiology and Immunology, Pathology Department, University of Utah, Salt Lake City, UT, United States; ^4^Department of Environmental Sciences, University of California, Riverside, CA, United States

**Keywords:** lytic phage, antibiotic resistance, *Klebsiella pneumoniae*, NDM-1, polyvalency

## Abstract

Bacteriophage KL-2146 is a lytic virus isolated to infect *Klebsiella pneumoniae* BAA2146, a pathogen carrying the broad range antibiotic resistance gene New Delhi metallo-betalactamase-1 (NDM-1). Upon complete characterization, the virus is shown to belong to the *Drexlerviridae* family and is a member of the Webervirus genus located within the (formerly) T1-like cluster of phages. Its double-stranded (dsDNA) genome is 47,844 bp long and is predicted to have 74 protein-coding sequences (CDS). After challenging a variety of *K. pneumoniae* strains with phage KL-2146, grown on the NDM-1 positive strain BAA-2146, polyvalence was shown for a single antibiotic-sensitive strain, *K. pneumoniae* 13,883, with a very low initial infection efficiency in liquid culture. However, after one or more cycles of infection in *K. pneumoniae* 13,883, nearly 100% infection efficiency was achieved, while infection efficiency toward its original host, *K. pneumoniae* BAA-2146, was decreased. This change in host specificity is reversible upon re-infection of the NDM-1 positive strain (BAA-2146) using phages grown on the NDM-1 negative strain (13883). In biofilm infectivity experiments, the polyvalent nature of KL-2146 was demonstrated with the killing of both the multidrug-resistant *K. pneumoniae* BAA-2146 and drug-sensitive 13,883 in a multi-strain biofilm. The ability to infect an alternate, antibiotic-sensitive strain makes KL-2146 a useful model for studying phages infecting the NDM-1+ strain, *K. pneumoniae* BAA-2146.

**GRAPHICAL ABSTRACT:**
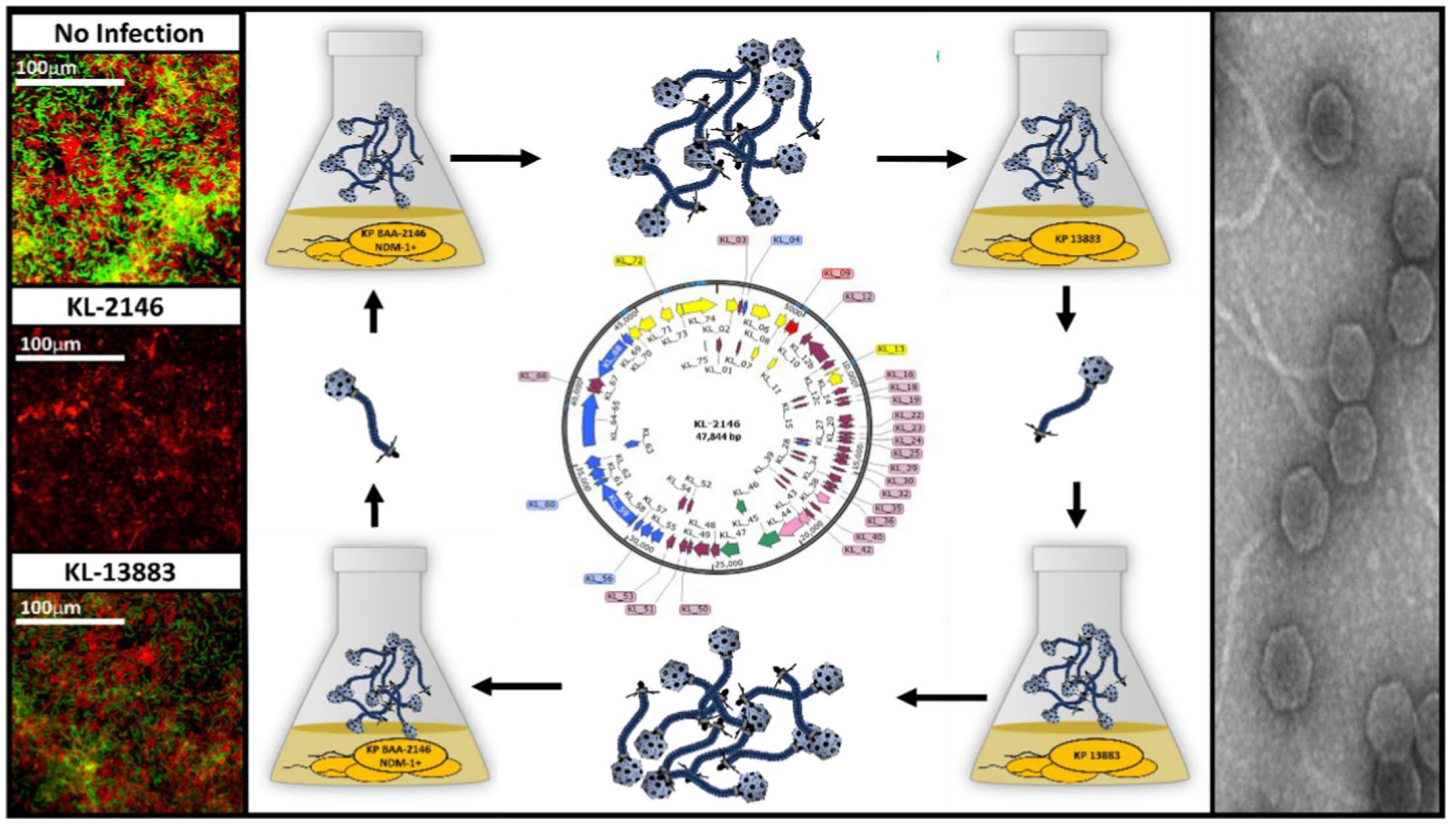

## 1. Introduction

Bacteriophages, viruses that infect bacteria, are presently of significant interest to researchers concerned with the proliferation of antibiotic-resistant genes in the environment and the increasing need for alternative therapies (Domingo-Calap and Delgado-Martínez, 2018). This interest is justified, in part, due to the aggressively antimicrobial nature of bacteriophages, which are responsible for 20% of the bacterial turnover on Earth (Suttle, 2007). Bacteriophages destroy antibiotic-resihreferstant bacteria with the same efficacy as any other host and are self-propagating, increasing their numbers exponentially after a low initial dose. Bacteriophage therapy has been proven effective in treating life-threatening infections where traditional antibiotics have failed (Huang et al., 2022) with phage-based products commercially available that include brand names such as PhageGuard (for the control of Listeria) or Intralytix (for treatment of *E. coli* infections). Despite such examples and increasing support from researchers and doctors who recognize the potential of phage therapy, obstacles to its widespread implementation persist (Sulakvelidze et al., 2001; Loc-Carrilloand and Abedon, 2011; Brüssow, 2019). Limitations primarily include (1) difficulty in getting phage to the relevant host sites at reasonable efficiency, (2) the large numbers of virions that may be required to have an effect, and (3) a generally limited virus host range (Sulakvelidze et al., 2001; Duckworth and Gulig, 2002).

The last obstacle, bacteriophage host specificity, is also considered one of the advantages of phage therapy in that it is possible to target specific problem bacteria while sparing other, perhaps helpful, bacterial species. However, limiting phage infectibility to a single species also becomes a challenge when one needs to apply phage therapy to multiple pathogenic bacterial species or multiple bacteria co-present in environmental applications (e.g., against biofilms or filamentous bulking). Even within the same species, bacterial variations can preclude the efficacy of phage treatment, necessitating precise diagnosis along with large existing phage banks (Nilsson, 2014). For applications where a broad host range is advantageous, such as when confronted with diverse community structures present in biofilms, the search for polyvalent bacteriophages (phages that can infect hosts of different genera or species) is on the rise.

Although biofilms can form from a single species of bacteria, they are most often composed of a diverse range of organisms that include different species of bacteria, algae, fungi, and protozoa (Wimpenny et al., 2000; Balcázar et al., 2015). In complex heterogeneous communities such as this, biofilms are most effective in providing a physical barrier for microbes against environmental stressors like antibiotics and phage predation (Hall and Mah, 2017). Cells within biofilms also tend to be less metabolically active, rendering many antimicrobials ineffective (Stewart, 2015). These are just a few reasons bacterial infections and engineering challenges resulting from biofilm formation are so difficult to address. With antibiotics and disinfectants proving limited in their ability to clear biofilms, bacteriophage present a promising therapeutic alternative.

This manuscript presents a lytic bacteriophage, KL-2146, isolated from a wastewater treatment plant (WWTP) in Salt Lake City, Utah. Its host, *Klebsiella pneumoniae BAA-2146* (Hudson et al., 2014), was originally isolated from a US hospital patient in 2010 who had recently received medical care at a clinic in India. *K. pneumoniae* BAA-2146 carries multiple antibiotic resistance genes (ARG) on multiple plasmids, including the broad range ARG New Delhi Metallo-β-lactamase 1 (NDM-1), making it resistant to most known antibiotics (Yong et al., 2009). Phage KL-2146 was determined to be polyvalent in that it can infect another *Klebsiella* host species, *K. pneumoniae* 13,883, which is antibiotic-sensitive. It was also demonstrated to effectively disrupt biofilms possessing multiple *Klebsiella* strains in contrast to another isolated non-polyvalent phage species capable of infecting only a single strain.

Detailed studies of potentially useful bacteriophages benefit from experimentation with gene knockouts, substitutions, fusions, tags, and targeting endonucleases, e.g., CRISPR (Chen et al., 2019). Because these methods primarily involve the use of antibiotics as selective markers in the host strain, conducting these experiments in bacterial strains that are not susceptible to antibiotics is very difficult. For this reason, and because of the inherent risks in handling such bacteria, the ability of a phage such as KL-2146 to infect multiple hosts (polyvalence), including antibiotic-sensitive hosts, is a valuable feature. This report provides the first example of polyvalence in a phage infecting an NDM-1 positive *Klebsiella* bacterial strain. It also discusses an observed switching in host infection efficiency between the host strains *K*. *pneumoniae* BAA-2146 and *K*. *pneumoniae* 13,883.

## 2. Materials and methods

### 2.1. Host characteristics and growth procedures

*Klebsiella pneumoniae* ATCC# BAA-2146, possessing NDM-1 (Hudson et al., 2014), is a Gram-negative, non-motile, encapsulated, rod-shaped bacterium. This strain is resistant to most known antibiotics (Yong et al., 2009), with multiple narrow and broad-range antibiotic resistance genes present on several large plasmids. The second potential host that KL-2146 is tested against is *K. pneumoniae* subsp. *pneumoniae* ATCC# 13883 (Daligault et al., 2014). This strain possesses the same basic structural features as the NDM-1+ strain, is cultured using the same methods, and has no known antibiotic resistances. Other strains used for determining the polyvalence of phage KL-2146 include *K. pneumoniae* ATCC# 35657 (Ayu et al., 2014), *Klebsiella oxytocca* CCUG# 15788 (Singh et al., 2016), which is a close relative of *K. pneumoniae,* and clinical isolates of *K. pneumoniae* (19C5A and 19C6A) obtained from Dr. Matt Mulvey’s lab at the University of Utah (Mulvey and Simor, 2009). Growth curves of *K. pneumoniae* BAA-2146 and *K*. *pneumoniae* 13883 were determined experimentally from single colony isolations grown overnight in LB (Miller, 1972) at 37°C. Newly 1:100 inoculated cultures were shaken (190 RPM) at 37°C with 1 mL of the sample taken for spectrophotometric measurements (OD_595_) every 10 min. Colony forming units (CFU/mL) at exponential growth was determined by chilling samples on Ice for 10 min, serial dilution in LB, and plating on LB + 0.7% agar. Detailed growth curves were not established for the other potential hosts, which did not yield plaques from phages used in this study.

### 2.2. Phage isolation, propagation, and physical characterization

**Figure 1:**
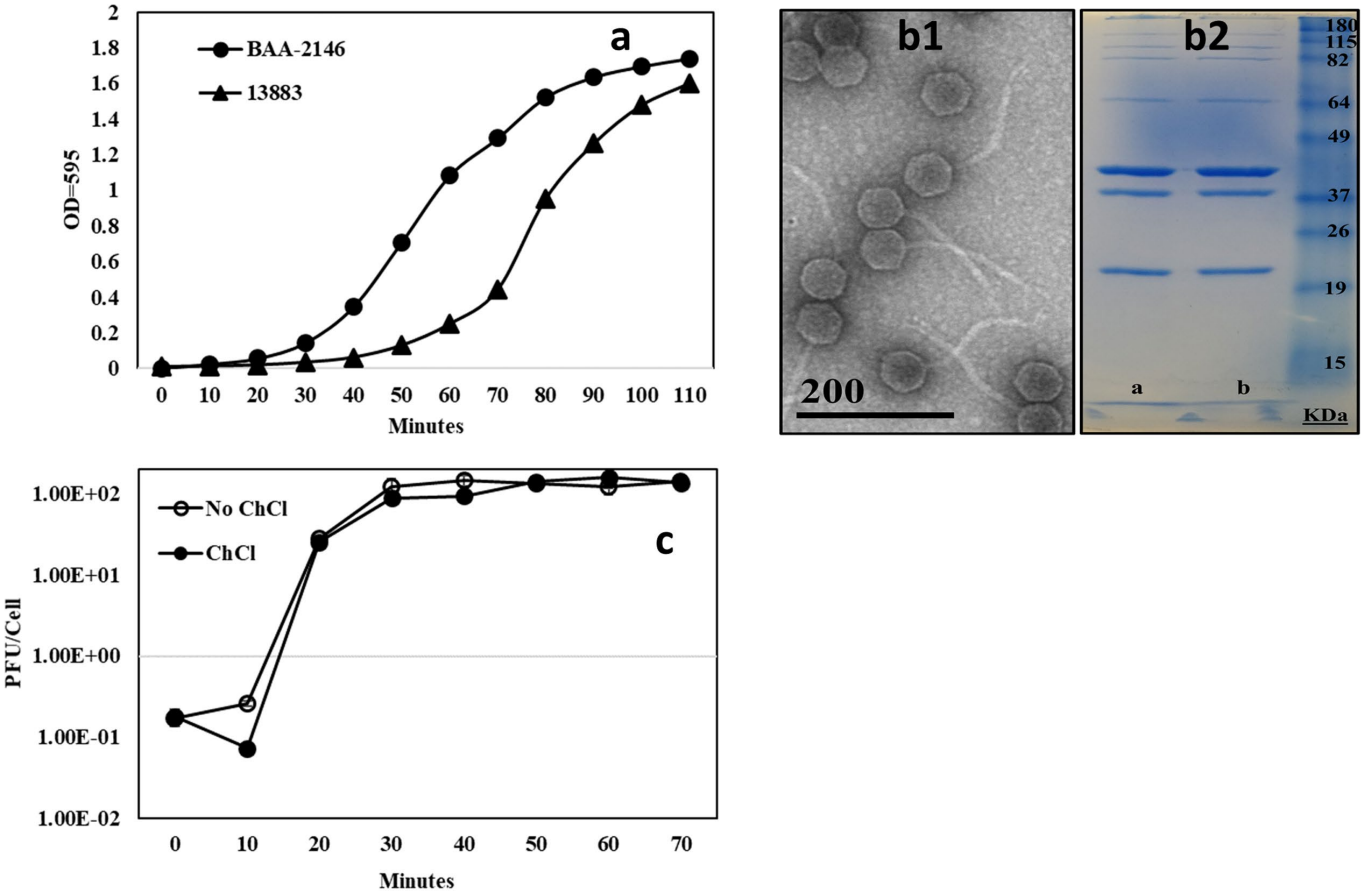
**(A)** [*n* = 3] Growth curve comparison of *K. pneumoniae* 13,883 and the NDM1+ strain *K. pneumoniae* BAA-2146. CFU/mL at exponential phase of growth indicated, **(B1)** TEM of bacteriophage KL-2146, **(B2)** 13% acrylamide SDS-PAGE gel of CsCl purified phage KL-2146 isolated from *K. pneumoniae* BAA-2146 and *K. pneumoniae* 13,883 (respectively). No difference in band pattern was observed, and **(C)** [*n* = 3] One-step growth curve of phage KL-2461 infecting *K. pneumoniae* BAA-2146.

Phage KL-2146 was isolated from activated sludge obtained from a wastewater treatment plant (Salt Lake City, Utah) by infection of *K. pneumoniae* BAA-2146 utilizing methods in sequential filtration and centrifugation previously described (Delfan et al., 2012; Bhattacharjee et al., 2017; Hockett and Baltrus, 2017). A single plaque was thusly isolated and propagated by infection of the same hosts in liquid culture (Earnshaw et al., 1976). Phage lysates from cultures were shaken with chloroform and centrifuged for 20 min at 6000 g. The cleared lysate was then titered for downstream infections of larger (500 mL) cultured using an MOI = 10. Upon lysis, these cultures were concentrated by overnight centrifugation (15,302 g, JA 16.25 Beckman rotor) and resuspended in SMG buffer (50 mM Tris–HCl pH = 7.5, 100 mM NaCl, 10 mM MgSO_4_, 0.01% gelatin). Purification of phages was performed by CsCl isopycnic centrifugation (Earnshaw et al., 1976) using protocols described in Gilcrease et al. (2020). Phage concentrate was layered onto a step gradient of SMG + 10% Sucrose, SMG + 1.4 g/mL CsCl, and SMG + 1.6 g/mL CsCl in an SW41 Ultraclear™ centrifuge tube for SW41 Beckman rotors. These preparations were then centrifuged at 220,000 g for 2 h at 20°C. The band containing phage, located at the 1.4/1.6 CsCl interface, was extracted with a syringe and dialyzed against SMG buffer using a Spectra/ Por™ 7 50,000 MWCO dialysis membrane. DNA was extracted for sequencing using the Norgen Phage DNA Isolation kit (cat# 46800). Proteins of mature virions were visualized with 12% acrylamide SDS-PAGE gels using the Mini-PROTEAN II gel system, a protein sizing standard (Thermofisher Benchmark Protein Ladder. Cat#10748010), and procedures described in Eppler et al. (1991). Basic phage structural morphology was determined by negative staining transmission electron microscopy (TEM, model JEOL JEM-1400) using methods described in Gilcrease et al. (2020). A phage lacking polyvalence was also isolated to compare phage infection efficiencies toward a multi-strain biofilm. It was isolated on *K. pneumoniae* 13,883 using the previously described methods and only makes plaques on that strain. This non-polyvalent strain, called phage KL-13883, was not further characterized in this study. Detailed one-step growth curve analysis for phage KL-2146 [*n* = 3] was conducted by infection of 100 mL host culture at exponential growth phase (OD_595_ = 0.5) at 37°C, with a multiplicity of infection (MOI) of 10 phages per colony forming unit (CFU). This higher-than-typical MOI ensures that all bacteria are infected (Nale et al., 2021) and is the same MOI used for large-batch phage purification. This produces total lysis after the first round of infection and allows ease of determining the Plaque Forming Units (PFU)/cell using the CFU/mL known at the time of infection from previous bacterial growth analysis. Two 1 mL samples were then taken every 10 min, one of which was vortexed with 100 μL chloroform. Both were then centrifuged for 5 min at 6000 *g,* transferred to fresh Eppendorf tubes, serially diluted, and tittered using the agar overlay method (Hockett and Baltrus, 2017). Growth curves were plotted by dividing the PFU yield by the bacteria concentration in culture, which was determined experimentally to be 1.2 × 10^8^ CFU/mL at OD_595_ = 0.5 (Figure 1) to obtain PFU/cell. In other experiments where infection efficiency is compared, an MOI =0.026 was used, and samples were taken after only 70 min of infection for comparative analysis. This is when detailed growth curves suggest maximum lysis has occurred after the 1st round of replication in culture. Plaque forming units (PFU) were visualized and quantified using the agar overlay method.

### 2.3. Phage KL-2146 genome sequencing, assembly, and annotation

After extraction of viral DNA, quality was determined by 16S rDNA PCR amplification (to ensure no host DNA contamination) and quantified by spectrophotometry (Thermo Scientific. NanoDrop 1,000). An Illumina TruSeq DNA Library preparation (450 mean insert size) with UDI was performed, followed by Illumina MiSeq 150-cycle paired-end sequencing at the University of Utah HighThroughput Genomics Core Facility. Approximately 29, 303 paired-end reads were trimmed based on a quality score (Phred score) of 28 and *de novo* assembled using software (Geneious™). GeneMarkS (Besemer et al., 2001) predicted genes were used to search the NCBI non-redundant (nr), conserved domain, COG, and InterPro databases and were annotated using Blast2GO 2.5.0 (Conesa et al., 2005) with accompanying manual curation. ARAGORN (Laslett and Canback, 2004) and tRNAScanSE (Schattner et al., 2005) were used for detecting tRNA genes. Phage KL-2146_mod_ (KL-2146 re-isolated on the 13,883 strain) was prepared, *de novo* assembled, and analyzed using the same procedures after trimming 33,212 paired-end reads. Comparative analysis of sequence data for phages KL-2146 and KL-2146_mod_ was done using Snapgene™. Taxonomy was informed using the Basic Local Alignment Search Tool (BLAST) of the 959 bp major head gene (H1O04_gp48) against NIH sequence databases.

### 2.4. Switching of host specificity and examining polyvalence

To investigate polyvalence and the observed switching of host specificity, phages were separately isolated from KL-2146 infections of different *Klebsiella* hosts. Phage KL-2146 that was propagated on *K. pneumoniae* BAA-2146 from individual plaques is referred to as KL-2146. KL-2146_mod_ was made by propagating KL-2146 through *K. pneumoniae* 13,883. In making KL-2146_mod_, *K. pneumoniae* 13,883 was used for the isolation of an individual plaque of KL-2146 (MOI = 0.26). This plaque was allowed to diffuse into 1 mL LB overnight and then used to infect 50 mL of *K. pneumoniae* 13,883 to produce a titered stock. This stock was later used for making larger, cesium-purified phage preparations in the 13,883 strain. In subsequent experiments, the purified stock of KL-2146_mod_, which infects *K. pneumoniae* 13,883 with nearly 100% infection efficiency, was used to infect its original host, *K. pneumoniae* BAA-2146. Similar to the KL-2146_mod_ isolation, plaques from the resulting infection were picked and used to reinfect the original *K. pneumoniae* BAA-2146 culture. The new phage stock is called KL-2146_new_. The PFU/mL values given for these experiments are averages taken from [*n* ≥ 3] experimental repeats.

### 2.5. Infection of multi-strain biofilm

Heterogeneous biofilms containing the two primary *K. pneumoniae* strains were grown using two different methods. In the first method, biofilms were grown on glass microscope slides using methods adapted from Bhattacharjee et al. (2015). To compensate for the slower growth rate inherent to 13,883, more of this strain was used to inoculate the cultures than the faster-growing BAA-2146 strain. Hence, 25 mL of LB was inoculated with 1:100 overnight cultures of *K. pneumoniae* 13,883 and 1:250 *K. pneumoniae* BAA-2146 in a 50 mL conical tube. 75 mm × 25 mm glass slides were inserted into culture tubes and incubated in the upright position overnight at 37°C. Media was replaced by carefully transferring the slides to a tube containing fresh LB every 24 h for 4 days.

At this time, bacteriophages KL-2146, KL-2146_mod_, KL-2146_new_, and KL-13883 were added to 25 mL of fresh LB to a final concentration of 1×10^7^ in four separate 50 mL conical tubes. A fifth tube was prepared without bacteriophages as a control. Glass slides containing biofilm were submerged in the media + phage for 12 h at 37°C. Slides were then washed by dipping them carefully in PBS solution. For CFU quantification of the two strains present in biofilm after 6 days, just prior to staining, samples of biofilm were removed for CFU analysis by scraping across the center of the slide using a 1 cm wide metal spatula. The biofilm substance on the spatula was then transferred to 1 mL of ice-cold LB in a 1.5 mL Eppendorf tube. The samples were serially diluted and plated onto two agar+ LB plates, one with 50 μg/mL ampicillin and the other with “no-drug.” Plates were incubated overnight at 37°C, and colonies were counted the next day.

Immediately after the above sampling of biofilm from the slides, slides were dipped into a methanol bath for fixation, air dried, and stained using a Gram staining kit (Brown and Brenn Cat# KTBBR) following the instructions provided therein. Photographs of the biofilm were taken using a light microscope at 100X magnification.

For the second method, biofilms were grown using a custom-made drip flow reactor. Glass microscope slides were seeded for 6 h at room temperature with 25 mL of a 1:100 host culture in 50 mL conical tubes. Slides were then placed at a slight angle into a drip flow reactor so that LB media was allowed to drip over slides at a rate of (1.5 mL/h) for 4 days using a peristaltic pump. Slides were then washed in PBS and stained using a Baclight Bacterial Viability kit (Invitrogen Cat# L7012) following instructions contained in the manual. Biofilms were then visualized using a Nikon Widefield microscope at 60X magnification.

### 2.6. Transmission electron microscopy

For TEM visualization, 1×10^11^ PFU/mL of CsCl purified phages were dialyzed in SMG buffer. 3 μL of this mixture was laid onto Formvar 200 Mesh Copper grids that had been subjected previously to a 20 s plasma ionization treatment. After 1 min, the excess phage solution was removed with filter paper. 3 μL of 1% uranyl acetate was added to the grid, left for 45 s, and extracted with filter paper. All negatively stained TEM images were obtained using a TEM model JEOL JEM-1400.

### 2.7. Statistical analysis

The averages of at least 3 technical repeats were taken for CFU and PFU values given, with standard deviation bars or error percentages provided. To determine statistical differences in the number of plaques in growth curve results, one-way analysis (ANOVA, Excel) was performed between the two different data sets. Significance was determined at an alpha level of 0.05 (*p* ≤ 0.05) and is the standard set for all [*n* ≥ 3] data that is presented.

## 3. Results

### 3.1. Structural and growth characteristics of isolated lytic phages and their hosts

In all experiments, the bacteria strain *K. pneumoniae* 13883 was observed to grow slower than BAA-2146, lagging behind BAA-2146 in reaching exponential phase by ~30 minutes, as shown in the growth cycle in Figure 1A. *K. pneumoniae* 35657, *K. oxytocca*, and sequenced clinical isolates *K. pneumoniae* 19C5A and 19C6A had growth rates similar to *K. pneumoniae* BAA-2146 (data not shown).

All of the KL-2146 isolations used in this study (KL-2146, KL-2146_mod_, and KL-2146_new_) share identical virus morphology as determined by negative stain TEM; hence only images of KL-2146 are provided (Figure 1B1). In TEM, KL-2146 was structurally observed to be a member of the Drexlerviridae family (formerly *Siphoviridae*) possessing an isometric head approximately 60 nm in diameter and a ~150 nm long, filamentous, non-contractile tail. SDS-PAGE analysis of purified KL-2146 virions (Figure 1B2) showed seven observable structural proteins. KL-2146 and KL-2146_mod_ (the same phage isolated on different strains of *K. pneumoniae*) do not show any differences in the size or the total number of protein bands in SDS-PAGE. Phage KL-2146 is shown in one-step growth curves to have a burst size of approximately 142 pfu/cell with a generation time of about 30 min, as shown in the phage growth cycle in Figure 1C. It is a virulent (or lytic) phage based on clear plaque formation and a lack of genes found in sequence analysis that might suggest a lysogenic lifestyle present in the assembled sequence, such as integrases or ParA/B genes (Emerson et al., 2012).

### 3.2. Phage genome sequencing and analysis

To characterize the genetic composition of KL-2146 and to conduct phylogenetic analysis, whole genome sequencing of the purified phage was conducted *via* Illumina, with the complete genome deposited in GenBank under the accession number MN379832. The KL-2146 genome is 47,844 bps long and 50.8% G + C with circular permutation. Seventy-four encoded proteins are annotated in the genome, including a recognizable large terminase, major capsid protein, several tail proteins including tail tape measure, lysis proteins, and nucleic acid metabolism proteins including DNA helicase, primase, single-strand DNA binding protein, exonuclease, and DNA methylase. Genome sequence analysis revealed phage KL-2146 to be a member of the *Webervirus* genus within what had previously been described as the T1-like cluster of tailed phages (Casjens and Grose, 2016) or *Tunavirinae*.

### 3.3. Phage infectivity to its parent host and other strains

**Figure 2:**
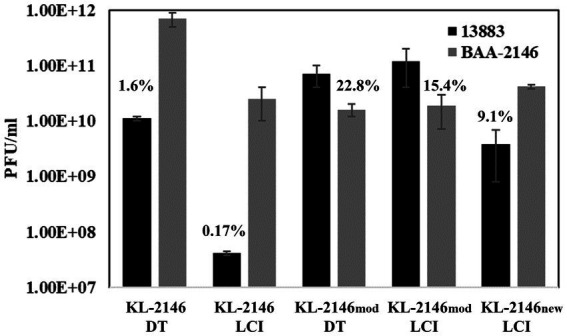
[*n* = 3] Infections of *K. pneumoniae* 13,883 and BAA-2146 using cesium-purified phage KL-2146 (original isolation made from infection of the BAA-2146, NDM-1+ strain) and KL-2146_mod_ (phage stock made from infection of the 13,883 strain). KL-2146_new_ is lysate from phage KL-2146_mod_ propagated on BAA-2146. This tests the ability of KL-2146_mod_ to re-acquire host specificity to BAA-2146. DT = Direct titration onto host lawns. LCT = titration after 70 min of Liquid Culture Infection. Percentage values are from PFU comparisons of titers on [new strain/parent strain]. Error bars indicate standard deviation.

KL-2146 was shown to be highly infectious to its host BAA-2146 (the original host from which it was isolated), with complete lysis of culture visible after 30 min of MOI = 10 infection or 90 min of MOI = 0.026 infection. To assess polyvalent properties, phage KL-2146 infectivity was tested using a variety of *K. pneumoniae* strains phylogenetically similar to BAA-2146 (Supplementary Table 1). Using the plaque overlay method, a faint clearing of bacteria was noted in spot tests on *K. pneumoniae* 35,657 at low dilutions, but no plaques were produced. No faint clearing from concentrated lysates or plaques was observed upon attempts to infect strains of *K. pneumoniae* clinical isolates 19C5A, 19C6A, or the *K. oxytocca* strain. Of the strains tested, only *K. pneumoniae* strain 13,883 showed clear evidence of infection (plaque formation). KL-2146 is thusly considered a polyvalent phage in that it can infect different strains, or serovars, *K. pneumonia*. However, plaque titers of KL-2146 on *K. pneumoniae* 13,883 produced significantly fewer PFUs (approximately 1.6%) than when titered on the original host *K. pneumoniae* BAA-2146. Seventy-minute infections of BAA-2146 and 13,883 were then conducted in parallel using the original cesium chloride density gradient purified phage KL-2146 that had been isolated using BAA-2146 as a host. In these liquid culture infections, those phages propagated on BAA-2146 were shown when infecting 13,883 to produce an even lower yield (0.17%) compared with infections of BAA-2146 at *t* = 70 min (Figure 2). The difference in observed yield between direct plaque assay of phage and plaque assays conducted after infection of liquid culture remains unclear, but may be due to (1) more favorable exposure conditions (e.g., more time for attachment) under conditions of direct titration or (2) differences in initial burst size would not be observable on direct titration since lower (or stunted) phage yields from the first round of infection will produce the same number of plaques overnight as higher/normal yields. To assess whether or not KL-2146 infection efficiency would remain stunted after isolation from 13,883 or become acclimated to the new host and infect normally, plaques taken from the less efficient 13,883 infections were further propagated on 13,883 in liquid culture. After concentration and purification, the resulting phage (now called KL-2146_mod_) was found to have acquired normal infection efficiency against this new 13,883 host strain, with PFU yields similar to KL-2146 infections of BAA-2146 (Figure 2). The KL-2146_mod_ (with 13,883 as its new host) phage that had been thusly “acclimated” to host 13,883 was shown later to have reduced specificity towards its original host, BAA-2146. This was observed in experiments where phage KL-2146_mod,_ again purified from infections of 13,883, was seen to produce low phage yields (~15.4%) when infecting the original BAA-2146 strain (Figure 2). In order to determine whether the specificity of KL-2146_mod_ towards the BAA-2146 strain can be re-acquired after having been lost, the lysate from phage KL-2146_mod_ was titered on the original host BAA-2146. Plaques were isolated and further propagated on the BAA-2146 host, with the resulting stock now called phage KL-2146_new_. This was then used to infect BAA-2146. After 70 min, PFU from infections of BAA-2146 with the KL-2146_new_ lysate indeed shows a reversal to total infectability of the BAA-2146 strain (Figure 2).

### 3.4. Infections of a multi-strain biofilm

**Figure 3:**
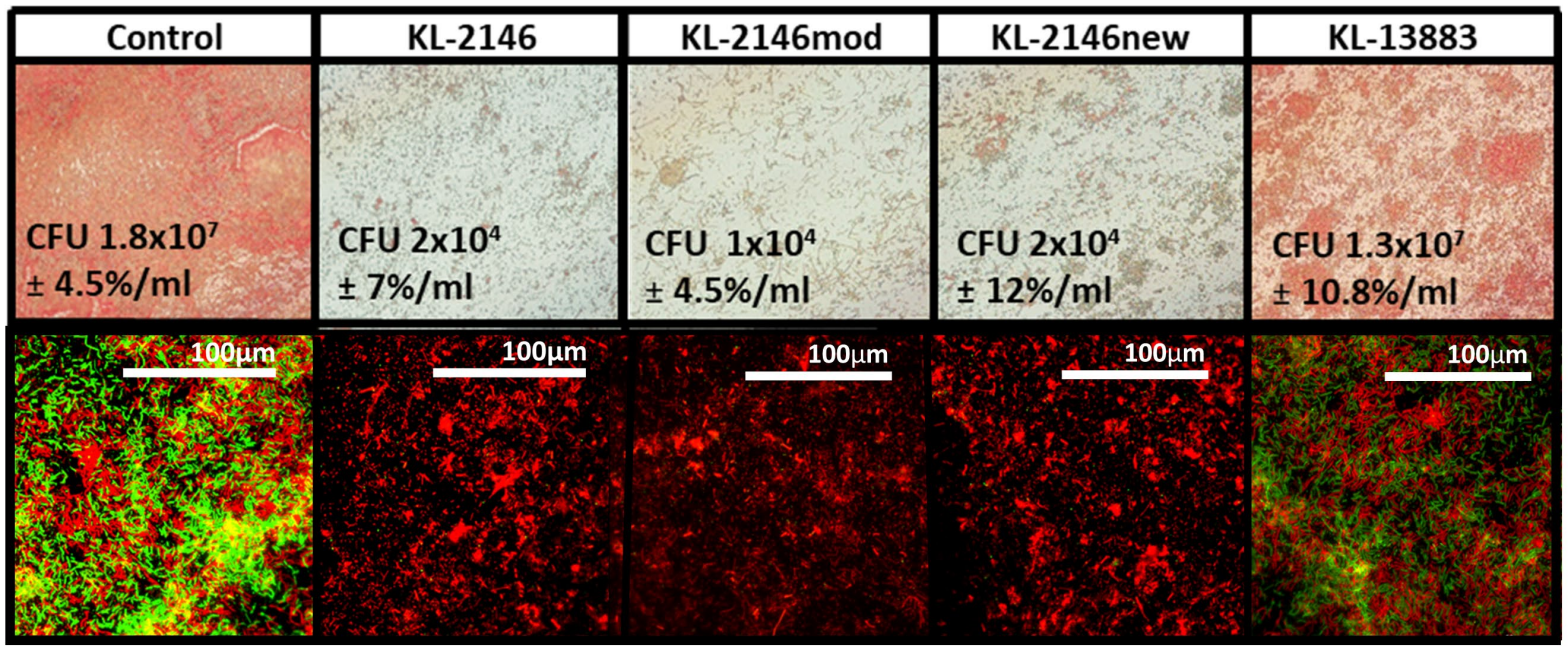
Six-day heterogenous biofilms of *K. pneumoniae* 13,883 and BAA-2146 were infected with 1×10^7^ phages/mL for 12 h. **Top row:** Slides seeded with bacteria and submerged fresh media (replaced daily) in were gram stained and visualized at 100X. **Bottom row:** 60X magnification of biofilm from drip flow reactor (same order as above) stained with BacLite. Green = live, Red = Dead. Biofilms disruption demonstrated on all slides except the control and after infection with the non-polyvalent phage KL-13883. CFU values [*n* = 3] are from approximately 6 cm^2^ sampled from slide surfaces resuspended in 1 mL LB. Error percentages are the standard deviation/CFU.

A heterogeneous biofilm composed of bacteria from two separate strains of *K. pneumoniae* was constructed to demonstrate the comparative efficiencies of polyvalent virus infection versus non-polyvalent infection when two host strains are present. Biofilms were developed on microscope slides using both immersion and drip flow methods and visualized using a Gram Stain and Baclight Viability Kit, respectively. Biofilm heterogeneity was confirmed by counting CFU from the uninfected control on ampicillin plates (1 × 10^7^/mL ± 3×10^6^) for BAA-2146 and drug-free plates (1.8 × 10^7^ ± 2×10^6^) for the total number of bacteria. This suggests an approximate 1:1 ratio of BAA-2146 versus the antibiotic-sensitive strain 13,883, given the margins of error. In biofilm infection experiments, relative to the uninfected biofilm control, slides exposed to polyvalent phages were mainly cleared of biofilm due to infection of both strains (Figure 3). Backlight Live/Dead color labeling suggests that the comparatively few bacteria remaining on the slides after infection using the polyvalent phage are dead. There was no observable difference between polyvalent isolates KL-2146, KL-2146_mod_, and KL-2146_new_ regarding their efficacy in clearing the multi-strain biofilm. The biofilm slide exposed to the non-polyvalent phage, KL-13883, showed substantially less clearing than the uninfected control due to its ability only to infect the *K. pneumoniae* 13,883 strain. Surviving CFU values were nearly identical to uninfected control, implying a rapid replacement of 13,883 with the resistant bacterial strain. This demonstrates that the polyvalent strain is much more efficient at clearing multi-strain biofilms than a non-polyvalent strain, regardless of the less efficient initial infection of one host or the other.

## 4. Discussion

### 4.1. Phage structure and genome

**Figure 4:**
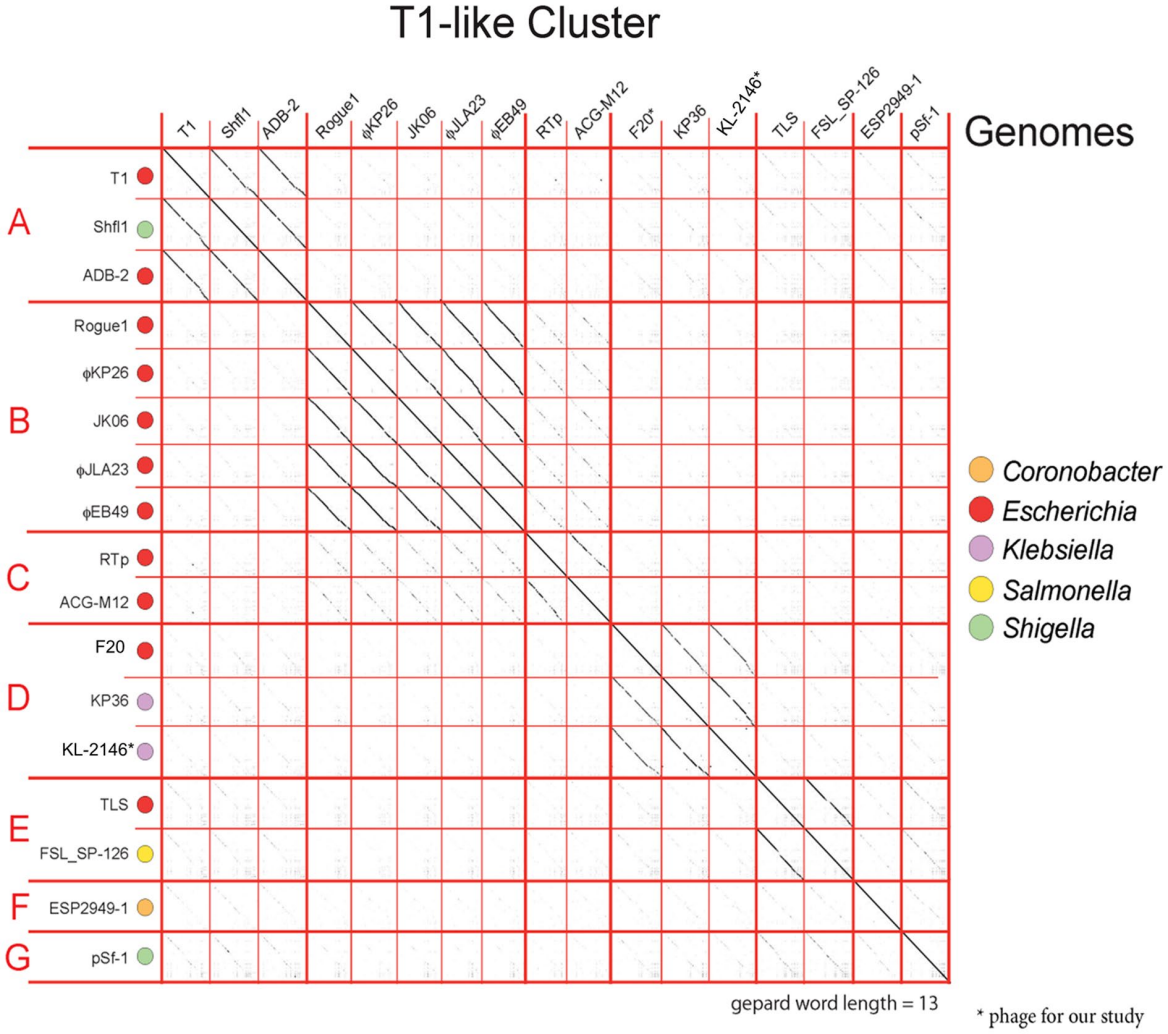
Dot matrix analysis of phage genome DNA alignments within the T1-Cluster of phages, constructed with a word length setting of 13 using Gephard (Krumsiek et al., 2007). The sequences, taken from GenBank, show the diversity of phages within the T1-cluster (Previously known an *Tunavirinae*). The continuity of diagonal lines indicates sequence similarity between phage genomes. The host genera are color coded. Subgroup D contains the nearest relatives of KL-2146 in the *Webervirus* genera.

At the time of this analysis, there were 133 complete genomes of *Weberviruses* in the public database, the majority of which fit within one of a dozen subgroups of the family previously called *Tunavirinae* (Korf et al. (2019) and our unpublished results). Figure 4 shows examples of seven of these subgroups, separated by bold horizontal lines. KL-2146 fits in the subgroup called “subcluster D” (Grose and Casjens, 2014) or the “*Webervirus*” genus (formerly the KP36like genus), all but one of which infects *K. pneumoniae* (Mishra et al., 2012; Hoyles et al., 2015). Among KL-2146’s closest relatives are bacteriophages Sanco (MK618657), MezzoGao (MF612072), GML-KpCol1 (MG552615), KPN N141 (MF415412), KL-2146PN1 (KT001920), KpKT21phi (MK278861), F20 (NC_043469.1) and NJR15 (MH633487), which were isolated in Texas, Maryland, Turkey, Korea, Ireland, Israel, Russia, and China, respectively (Nir-Paz et al., 2019; Richardson et al., 2019; Zurabov and Zhilenkov, 2019; Horváth et al., 2020). This closely related group of *Klebsiella* phages, all of which are >95% identical in nucleotide sequence, appears to be common and exceptionally widespread across the Earth. The KL-2146 genome reveals a variety of features that make it especially adaptable to host defenses. KL-2146, like many of the *Webervirus* phages, shares a notable lack of restriction sites such as HindIII, NsiI, NcoI, PaeI, and SnaBI (Hockett and Baltrus, 2017) and expresses its own DNA adenosine methyltransferase (KL-2146_2) suggesting a similar restriction evasion strategy to that of coliphage T1 (Auer and Schweiger, 1984). In addition, a well-studied klebsiella phage, phage KP36, encodes a tail-spike that has a capsule-targeting depolymerase (AXI63_gp50) that is capable of degrading the bacterial capsule and biofilms (Majkowska-Skrobek et al., 2016). The KL-2146 tail protein H1O04_gp67 is 90.32% identical to that protein.

### 4.2. Phage infection efficiency toward hosts

**Figure 5:**
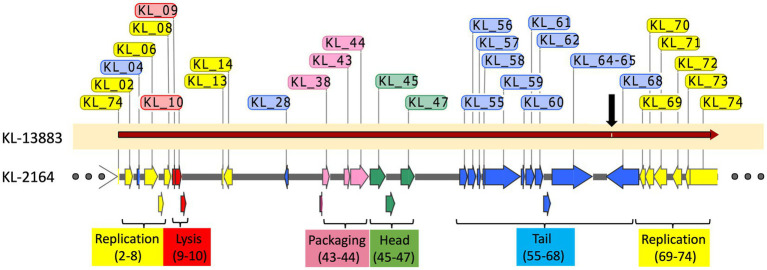
Alignment of KL-2146_mod_ (KL-2146 grown in *K. pneumoniae* 13,883) with the original KL-2146 strain (grown in *K. pneumoniae* BAA-2461). Hypothetical proteins are excluded. Only a single synonymous mutation (black arrow) observed.

The adaptable host specificity observed, whereby optimal efficiency of infection seemed to switch back and forth between hosts, is interesting and warranted further exploration. Bacteriophages evade host defenses and acquire new host specificities using a variety of strategies. These include the evolution of receptor-binding proteins in tail-spike or tail-fiber regions that allow recognition of new hosts, DNA methylation, and DNA sequence modification that protects against host restriction enzymes and CRISPR-acquired immunity (Labrie et al., 2010). Several attempts were thusly made to analyze the mechanism for this reversible switching in host infection efficiency from experimental data. (1) Restriction Avoidance: Restriction modification (RM) systems in bacteria are known to interfere with phage infection. In these systems, bacterial DNA is modified by methylation of specific sequences that prevent host endonuclease action on that sequence while allowing the targeted degradation of unmethylated foreign (e.g., viral) DNA that may enter the cell, thereby aborting infection (Gregory et al., 2010). In these cases, a phage may attach efficiently to a host but, lacking the host-derived DNA methylation pattern, will be unable to successfully complete infection. This can be overcome at low frequency when a rare infection event allows incoming viral DNA to escape DNA restriction and acquire the methylation pattern after a single round of infection, thereby producing progeny that are insensitive to host RM systems (Labrie et al., 2010). This is consistent with the observations of KL-2146 in Figure 2 regarding its back-and-forth host specificity and is the most likely explanation. The diversity of phages, restriction-modification systems, and host cells makes it difficult to provide a general frequency of restriction avoidance. Studies of this phenomenon using restriction-sensitive phage Lambda mutants suggest this typically happens at a lower frequency, 0.1–0.00001% (Arber, 1965; Rambach and Tiollais, 1974) than observed here for KL-2146 (1.6%). The robust KL-2146 restriction avoidance mechanism (i.e., lack of restriction sites in the genome) may increase the frequency of this avoidance beyond expectations, however. (2) Differences in Host Growth Rate: While in all infectivity experiments, the bacterial strain *K. pneumoniae* 13,883 was seen to grow slower than BAA-2146 (Figure 1A), this slower growth does not appear to affect infection yields of KL-2146_mod_ propagated in *K. pneumoniae* 13,883. This suggests that the slower growth of 13,883 compared with BAA-2146 is not a contributing factor in the observed lower phage yields from KL-2146 infections of 13,883. 3. Host Preference Altered by Mutation: These experiments, where samples are taken after 70 min of infection, suggest that acquisition of total infection efficiency occurs before or after the 2nd round of infection. It is unlikely that random mutation frequency could account for observable host switching within this period. Regardless, to determine whether KL2146_mod_ had acquired infection efficiency against *K. pneumoniae* 13,883 by means of mutation, whole genome sequence analysis of this isolation (a KL-2146_mod_ plaque grown in the 13,883 strain) was performed. DNA sequence alignments of this sequence with the KL-2146 genome showed only a single synonymous base-pair substitution that is not predicted to affect host specificity (Figure 5). In summary, the mechanism for host switching appears to be most similar to restriction avoidance, given its behavioral consistencies with what is known about the phenomenon and considering that other mechanistic candidates have been ruled out.

## 5. Conclusion

Phage KL-2146 is shown capable of infecting both strains BAA-2146 and 13,883 with equal efficiency only after a single step of inefficient propagation in the other strain, making it a potentially useful model system for the study of phage infection of an NDM-1+ pathogen. The study would have benefitted from including more polyvalent phages infecting NDM-1 for comparative study. It was observed, however, in isolation of these phages from wastewater that there were substantially fewer phages that infected the strain NDM-1 than the antibiotic-sensitive strain and even fewer that infected multiple strains, including the NDM-1 strain. Indeed, KL-2146 was the only successfully isolated polyvalent phage infector of *K. pneumoniae* NDM-1+. This is perhaps informative as to the abundance of this type of antibiotic-resistance bacteria present in the wastewater isolation source (Yahya et al., 2015).

The proliferation of *K. pneumoniae NDM-1+* strains and other broad-range antibiotic-resistant bacteria in selective reservoirs (such as hospitals and wastewater treatment plants) is an immediate concern, with multidrug resistance genes regularly making it into WWTP effluents and the larger ecosystem (Alexander et al., 2020). Bacteriophages are potentially an effective and ecologically safe antimicrobial tool, one of the few remaining capable of controlling such populations of bacteria in WWTPs. In addition, they can do so in a targeted manner, sparing those bacterial populations necessary for treatment processes. More study is needed to effectively exploit the antimicrobial nature of phages for actual use in such applications. Viral polyvalence is a property that not only allows infections of hosts outside of a single bacterial strain and enhanced removal of biofilms but also allows the detailed study and genetic manipulations of phages infecting these bacteria, particularly those with broad-range antibiotic resistances. This would otherwise be very difficult due to the lack of available selective markers (e.g., antibiotic resistance genes) usable in the resistant host. By exploiting polyvalence and conducting research efforts in an antibiotic-sensitive strain, modern DNA editing methods may be used to construct bacteriophages with enhanced infectivity, drug delivery capabilities, and DNA editing mechanisms such as CRISPR that can target and eliminate specific host genes (Gholizadeh et al., 2020). The study and characterization of phages like KL-2146 are thus an important addition to the emerging field of environmental phage therapy.

## Data availability statement

The datasets presented in this study can be found in online repositories. The names of the repository/repositories and accession number(s) can be found at: https://www.ncbi.nlm.nih.gov/genbank/, MN379832.

## Author contributions

AB conducted the initial isolation and sequencing of KL-2146. AB and EG conducted propagation/purification of phages and growth curve studies. SC, AB, and EG conducted phage genomic analysis. EG designed the polyvalence study and performed sequence and DNA analysis of KL-2146mod and structural analysis of both phages. EG and AB drafted the manuscript, which RG reviewed. All authors contributed to the article and approved the submitted version.

## Conflict of interest

The authors declare that the research was conducted in the absence of any commercial or financial relationships that could be construed as a potential conflict of interest.

## Publisher’s note

All claims expressed in this article are solely those of the authors and do not necessarily represent those of their affiliated organizations, or those of the publisher, the editors and the reviewers. Any product that may be evaluated in this article, or claim that may be made by its manufacturer, is not guaranteed or endorsed by the publisher.

## Abbreviations

NDM-1: New Delhi metallo-betalactamase-1
dsDNA: double-stranded Deoxyribonucleic acid
K: Klebsiell
a CFU: Colony forming units
PFU: Plaque Forming Units
NIH: National Institutes of Health
BLAST: Basic Local Alignment Search Tool
MOI: Multiplicity of infection
ATCC: American Type Culture Collection
CCUG: Culture Collection University Of Gothenburg
SMG: Saline Magnesium Gelatin
LB: Luria Broth
PCR: Polymerase chain reaction
TEM: Transmission electron microscopy
p: probability
n: number of repeats. SDS-PAGE
mod: modified
RM: Restriction modification

## References

[ref1] Alexander J. Hembach N. Schwartz T. (2020). Evaluation of antibiotic resistance dissemination by wastewater treatment plant effluents with different catchment areas in Germany. Sci. Rep. 10, 1–9. doi: 10.1038/s41598-020-65635-4 32488142 PMC7265433

[ref2] Arber W. (1965). Host-controlled modification of bacteriophage. Annu. Rev. Microbiol. 19, 365–378. doi: 10.1146/annurev.mi.19.100165.002053 5318444

[ref3] Auer B. Schweiger M. (1984). Evidence that Escherichia coli virus T1 induces a DNA methyltransferase. J. Virol. 49, 588–590. doi: 10.1128/jvi.49.2.588-590.1984, PMID: 6319759 PMC255502

[ref4] Ayu E. Suwanto A. Barus T. (2014). Klebsiella pneumoniae from Indonesian tempeh were genetically different from that of pathogenic isolates. Microbiol. Indonesia 8, 9–15. doi: 10.5454/mi.8.1.2

[ref6] Balcázar J. L. Subirats J. Borrego C. M. (2015). The role of biofilms as environmental reservoirs of antibiotic resistance. Front. Microbiol. 6:1216. doi: 10.3389/fmicb.2015.01216 26583011 PMC4628128

[ref500] Besemer J. Lomsadze A. Borodovsky M. (2001). GeneMarkS: a self-training method for prediction of gene starts in microbial genomes. Implications for finding sequence motifs in regulatory regions. Nucleic Acids Res. 29, 2607–2618. 11410670 10.1093/nar/29.12.2607 PMC55746

[ref7] Bhattacharjee A. S. Choi J. Motlagh A. M. Mukherjee S. T. Goel R. (2015). Bacteriophage therapy for membrane biofouling in membrane bioreactors and antibiotic‐resistant bacterial biofilms. Biotechnol. Bioeng. 112, 1644–1654. doi: 10.1002/bit.25574 25728819

[ref8] Bhattacharjee A. S. Motlagh A. M. Gilcrease E. B. Islam M. I. Casjens S. R. Goel R. (2017). Complete genome sequence of lytic bacteriophage RG-2014 that infects the multidrug resistant bacterium Delftia tsuruhatensis ARB-1. Stand. Genomic Sci. 12, 1–14. doi: 10.1186/s40793-017-0290-y 29270250 PMC5735904

[ref23] Brüssow H. (2019). Hurdles for phage therapy to become a reality—an editorial comment. Viruses 11:557. doi: 10.3390/v11060557 31212885 PMC6631134

[ref9] Casjens S. R. Grose J. H. (2016). Contributions of P2-and P22-like prophages to understanding the enormous diversity and abundance of tailed bacteriophages. Virology 496, 255–276. doi: 10.1016/j.virol.2016.05.022, PMID: 27372181 PMC4969182

[ref10] Chen Y. Batra H. Dong J. Chen C. Rao V. Tao P. (2019). Genetic engineering of bacteriophages against infectious diseases. Front. Microbiol. 10:954. doi: 10.3389/fmicb.2019.00954 31130936 PMC6509161

[ref11] Conesa A. Götz S. García-Gómez J. M. Terol J. Talón M. Robles M. (2005). Blast2GO: a universal tool for annotation, visualization and analysis in functional genomics research. Bioinformatics 21, 3674–3676. doi: 10.1093/bioinformatics/bti610, PMID: 16081474

[ref12] Daligault H. Davenport K. Minogue T. Bishop-Lilly K. Bruce D. Chain P.. (2014). Draft genome assembly of Klebsiella pneumoniae type strain ATCC 13883. Genome Announc. 2:e00939-14. doi: 10.1128/genomeA.00939-14 25291763 PMC4175198

[ref13] Delfan A. Etemadifar Z. Bouzari M. Emtiazi G. (2012). Screening of novel bacteriophage infection in pseudomonas putida isolated from potato disease. Jundishapur J. Microbiol. 5, 550–554. doi: 10.5812/jjm.3786

[ref14] Domingo-Calap P. Delgado-Martínez J. (2018). Bacteriophages: protagonists of a post-antibiotic era. Antibiotics 7:66. doi: 10.3390/antibiotics7030066, PMID: 30060506 PMC6163168

[ref15] Duckworth D. H. Gulig P. A. (2002). Bacteriophages. BioDrugs 16, 57–62. 11909002 10.2165/00063030-200216010-00006

[ref16] Earnshaw W. Casjens S. Harrison S. C. (1976). Assembly of the head of bacteriophage P22: x-ray diffraction from heads, proheads and related structures. J. Mol. Biol. 104, 387–410. doi: 10.1016/0022-2836(76)90278-3, PMID: 781287

[ref17] Emerson J. B. Thomas B. C. Andrade K. Allen E. E. Heidelberg K. B. Banfield J. F. (2012). Dynamic viral populations in hypersaline systems as revealed by metagenomic assembly. Appl. Environ. Microbiol. 78, 6309–6320. doi: 10.1128/AEM.01212-12 22773627 PMC3416638

[ref18] Eppler K. Wyckoff E. Goates J. Parr R. Casjens S. (1991). Nucleotide sequence of the bacteriophage P22 genes required for DNA packaging. Virology 183, 519–538. doi: 10.1016/0042-6822(91)90981-G, PMID: 1853558

[ref19] Gholizadeh P. Köse Ş. Dao S. Ganbarov K. Tanomand A. Dal T.. (2020). How CRISPR-Cas system could be used to combat antimicrobial resistance. Infect. Drug Resist. 13, 1111–1121. doi: 10.2147/IDR.S247271, PMID: 32368102 PMC7182461

[ref20] Gilcrease E. Williams R. Goel R. (2020). Evaluating the effect of silver nanoparticles on bacteriophage lytic infection cycle-a mechanistic understanding. Water Res. 181:115900. doi: 10.1016/j.watres.2020.115900, PMID: 32504909

[ref21] Gregory R. Saunders V. A. Saunders J. R. (2010). Rule-based simulation of temperate bacteriophage infection: restriction-modification as a limiter to infection of bacterial populations. Biosystems 100, 166–177. doi: 10.1016/j.biosystems.2010.02.010, PMID: 20211223

[ref22] Grose J. H. Casjens S. R. (2014). Understanding the enormous diversity of bacteriophages: the tailed phages that infect the bacterial family Enterobacteriaceae. Virology 468-470, 421–443. doi: 10.1016/j.virol.2014.08.024 25240328 PMC4301999

[ref24] Hall C. W. Mah T. F. (2017). Molecular mechanisms of biofilm-based antibiotic resistance and tolerance in pathogenic bacteria. FEMS Microbiol. Rev. 41:276301, 276–301. doi: 10.1093/femsre/fux010 28369412

[ref25] Hockett K. L. Baltrus D. A. (2017). Use of the soft-agar overlay technique to screen for bacterially produced inhibitory compounds. J. Vis. Exp.:55064. doi: 10.3791/55064, PMID: 28117830 PMC5352255

[ref26] Horváth M. Kovács T. Koderivalappil S. Ábrahám H. Rákhely G. Schneider G. (2020). Identification of a newly isolated lytic bacteriophage against K24 capsular type, carbapenem resistant Klebsiella pneumoniae isolates. Sci. Rep. 10, 1–11. doi: 10.1038/s41598-020-62691-8 32246126 PMC7125228

[ref27] Hoyles L. Murphy J. Neve H. Heller K. J. Turton J. F. Mahony J.. (2015). Klebsiella pneumoniae subsp. pneumoniae–bacteriophage combination from the caecal effluent of a healthy woman. PeerJ 3:e1061. doi: 10.7717/peerj.1061, PMID: 26246963 PMC4525690

[ref28] Huang Y. Wang W. Zhang Z. Gu Y. Huang A. Wang J.. (2022). Phage products for fighting antimicrobial resistance. Microorganisms 10:1324. doi: 10.3390/microorganisms10071324, PMID: 35889048 PMC9324367

[ref29] Hudson C. M. Bent Z. W. Meagher R. J. Williams K. P. (2014). Resistance determinants and mobile genetic elements of an NDM-1-encoding Klebsiella pneumoniae strain. PLoS One 9:e99209. doi: 10.1371/journal.pone.0099209 24905728 PMC4048246

[ref30] Korf I. H. Meier-Kolthoff J. P. Adriaenssens E. M. Kropinski A. M. Nimtz M. Rohde M.. (2019). Still something to discover: novel insights into Escherichia coli phage diversity and taxonomy. Viruses 11:454. doi: 10.3390/v11050454, PMID: 31109012 PMC6563267

[ref31] Krumsiek J. Arnold R. Rattei T. (2007). Gepard: a rapid and sensitive tool for creating dotplots on genome scale. Bioinformatics 23, 1026–1028. doi: 10.1093/bioinformatics/btm039, PMID: 17309896

[ref32] Labrie S. J. Samson J. E. Moineau S. (2010). Bacteriophage resistance mechanisms. Nat. Rev. Microbiol. 8, 317–327. doi: 10.1038/nrmicro2315 20348932

[ref33] Laslett D. Canback B. (2004). ARAGORN, a program to detect tRNA genes and tmRNA genes in nucleotide sequences. Nucleic Acids Res. 32, 11–16. doi: 10.1093/nar/gkh152, PMID: 14704338 PMC373265

[ref34] Loc-Carrilloand C. Abedon S. (2011). Pros and cons of phage therapy. Bacteriophage 1, 111–114. doi: 10.4161/bact.1.2.14590, PMID: 22334867 PMC3278648

[ref35] Majkowska-Skrobek G. Łątka A. Berisio R. Maciejewska B. Squeglia F. Romano M.. (2016). Capsule-targeting depolymerase, derived from Klebsiella KP36 phage, as a tool for the development of anti-virulent strategy. Viruses 8:324. doi: 10.3390/v8120324, PMID: 27916936 PMC5192385

[ref36] Miller J. H. (1972). Experiments in Molecular Genetics. Cold Spring Harbor Laboratory. Cold Spring Harbor, NY

[ref37] Mishra C. K. Choi T. J. Kang S. C. (2012). Isolation and characterization of a bacteriophage F20 virulent to Enterobacter aerogenes. J. Gen. Virol. 93, 2310–2314. doi: 10.1099/vir.0.043562-0, PMID: 22764320

[ref38] Mulvey M. Simor A. (2009). Antimicrobial resistance in hospitals: How concerned should we be? CMAJ 180, 408–415. doi: 10.1503/cmaj.080239 19221354 PMC2638041

[ref39] Nale J. Y. Al-Tayawi T. S. Heaphy S. Clokie M. R. (2021). Impact of phage CDHS-1 on the transcription, physiology and pathogenicity of a Clostridioides difficile ribotype 027 strain, R20291. Viruses 13:2262. doi: 10.3390/v13112262, PMID: 34835068 PMC8619979

[ref40] Nilsson A. S. (2014). Phage therapy—constraints and possibilities. Ups. J. Med. Sci. 119, 192–198. doi: 10.3109/03009734.2014.902878, PMID: 24678769 PMC4034558

[ref41] Nir-Paz R. Gelman D. Khouri A. Sisson B. M. FacKL-2146er, J Alkalay-Oren S.. (2019). Successful treatment of antibiotic-resistant, poly-microbial bone infection with bacteriophages and antibiotics combination. Clin. Infect. Dis. 69, 2015–2018. doi: 10.1093/cid/ciz222 30869755

[ref42] Rambach A. Tiollais P. (1974). Bacteriophage λ having eco RI endonuclease sites only in the nonessential region of the genome. Proc. Natl. Acad. Sci. 71, 3927–3930. doi: 10.1073/pnas.71.10.3927, PMID: 4530273 PMC434299

[ref43] Richardson R. W. Lessor L. O'Leary C. Gill J. Liu M. (2019). Complete genome sequence of Klebsiella pneumoniae siphophage sanco. Microbiol. Resour. Announc. 8:e01252-19. doi: 10.1128/MRA.01252-19, PMID: 31672751 PMC6953505

[ref44] Schattner P. Brooks A. N. Lowe T. M. (2005). The tRNAscan-SE, snoscan and snoGPS web servers for the detection of tRNAs and snoRNAs. Nucleic Acids Res. 33, W686–W689. doi: 10.1093/nar/gki366, PMID: 15980563 PMC1160127

[ref45] Singh L. Cariappa M. Kaur M. (2016). Klebsiellaoxytoca: an emerging pathogen? Med. J. Armed Forces India Dec. 72, S59–S61. doi: 10.1016/j.mjafi.2016.05.002, PMID: 28050072 PMC5192185

[ref46] Stewart P. S. (2015). Antimicrobial tolerance in biofilms. Microbiol. Spectr. 3, 3–3. doi: 10.1128/microbiolspec.MB-0010-2014 PMC4507308 26185072

[ref47] Sulakvelidze A. Alavidze Z. Morris J. G. (2001). Bacteriophage therapy. Antimicrob. Agents Chemother. 45, 649–659. doi: 10.1128/AAC.45.3.649-659.2001 11181338 PMC90351

[ref48] Suttle C. A. (2007). Marine viruses–major players in the global ecosystem. Nat. Rev. Microbiol. 5, 801–812. doi: 10.1038/nrmicro1750 17853907

[ref49] Wimpenny J. Manz W. Szewzyk U. (2000). Heterogeneity in biofilms. FEMS Microbiol. Rev. 24, 661–671. doi: 10.1111/j.1574-6976.2000.tb00565.x 11077157

[ref50] Yahya M. Hmaied F. Jebri S. Jofre J. Hamdi M. (2015). Bacteriophages as indicators of human and animal faecal contamination in raw and treated wastewaters from t unisia. J. Appl. Microbiol. 118, 1217–1225. doi: 10.1111/jam.12774, PMID: 25689071

[ref51] Yong D. Toleman M. A. Giske C. G. Cho H. S. Sundman K. Lee K.. (2009). Characterization of a new metallo-β-lactamase gene, blaNDM-1, and a novel erythromycin esterase gene carried on a unique genetic structure in Klebsiella pneumoniae sequence type 14 from India. Antimicrob. Agents Chemother. 53, 5046–5054. doi: 10.1128/AAC.00774-09, PMID: 19770275 PMC2786356

[ref52] Zurabov F. Zhilenkov E. (2019). Complete genome sequences of lytic polysaccharide degrading Klebsiella pneumoniae bacteriophages vB_KpnS_FZ10, vB_KpnP_FZ12, vB_KpnM_FZ14, and vB_KpnS_FZ41. Microbiol. Resour. Announc. 8, e00914–e00919. doi: 10.1128/MRA.00914-19 31558632 PMC6763647

