# Supplementary material for: A Klebsiella pneumoniae NDM-1+ bacteriophage: Adaptive polyvalence and disruption of heterogenous biofilms
Source: Front Microbiol. 2023 Feb 17;14:1100607. doi: 10.3389/fmicb.2023.1100607 (PMC9983693; doi:10.3389/fmicb.2023.1100607)

# A *Klebsiella Pneumoniae* NDM-1+ Bacteriophage: Adaptive Polyvalence and Disruption of

**Heterogenous Biofilms.**

**Eddie B. Gilcrease^1^, Sherwood R. Casjens^2,3^, Ananda Bhattacharjee ^4^, and Ramesh Goel^1*^**

1. Department of Civil and Environmental Engineering, University of Utah, Salt Lake City, UT. USA

2. School of Biological Sciences, University of Utah, Salt Lake City, UT. USA.

3. Division of Microbiology and Immunology, Pathology Department, University of Utah, Salt Lake City, UT. USA

4. Department of Environmental Sciences, University of California, Riverside, CA. USA

*Author to whom correspondence should be addressed:

RKG: phone: 801-581-6110; fax801-585-5477; email: ram.goel@utah.edu


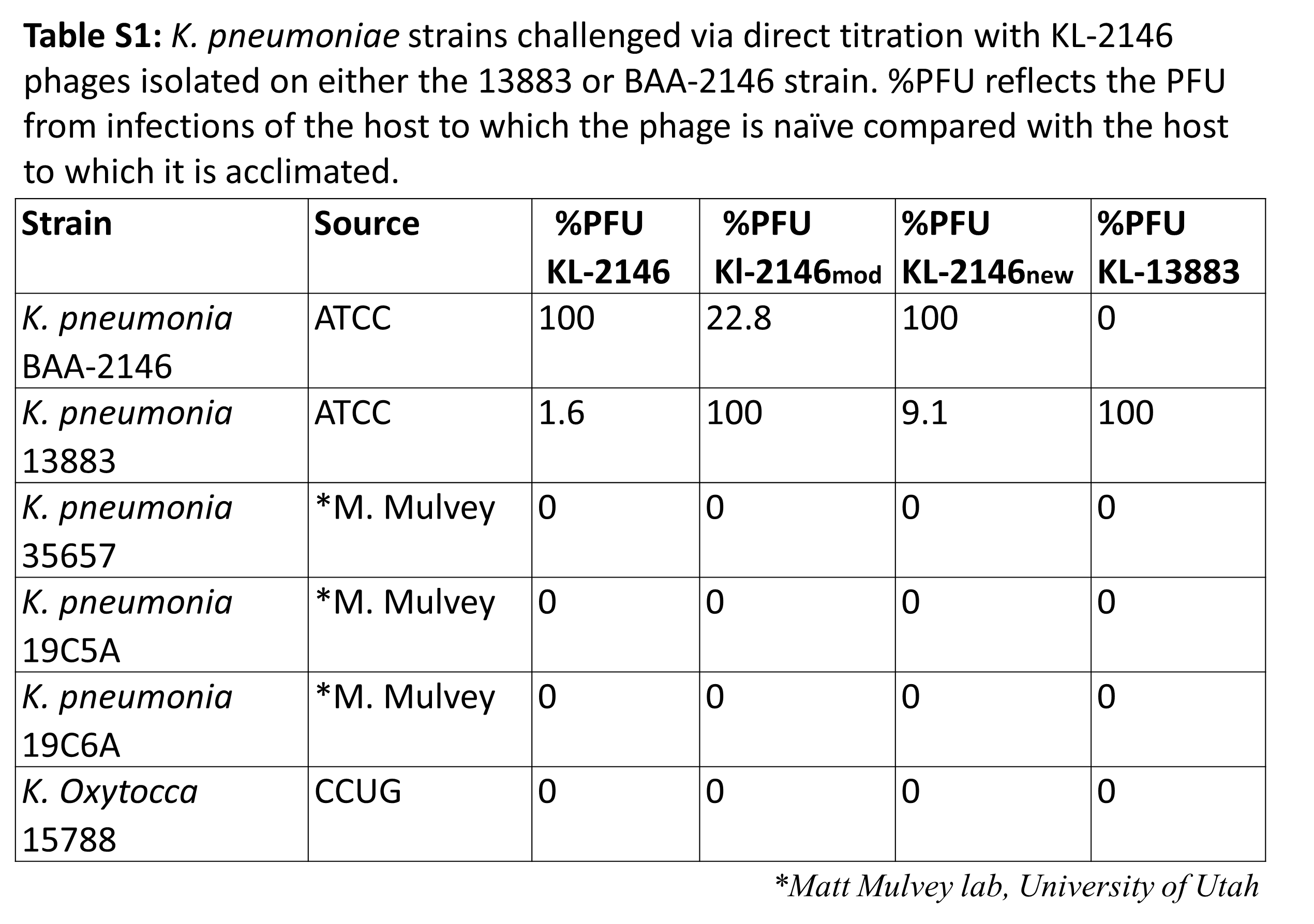

Supplement: Supplementary file 1 [file Table_1.DOCX]
